# How can we strengthen students’ social relations in order to reduce school dropout? An intervention development study within four Danish vocational schools

**DOI:** 10.1186/s12889-015-1831-1

**Published:** 2015-05-22

**Authors:** Liselotte Ingholt, Betina Bang Sørensen, Susan Andersen, Line Zinckernagel, Teresa Friis-Holmberg, Vibeke Asmussen Frank, Christiane Stock, Tine Tjørnhøj-Thomsen, Morten Hulvej Rod

**Affiliations:** Centre for Intervention Research in Health Promotion and Disease Prevention, National Institute of Public Health, University of Southern Denmark, Øster Farimagsgade 5A 2nd floor, DK-1353 Copenhagen K, Denmark; Centre for Alcohol and Drug Research, Aarhus University, Bartholins Alle 10, 3, DK-8000 Aarhus C, Denmark; Unit for Health Promotion Research, University of Southern Denmark, Niels Bohrs Vej 9, DK-6700 Esbjerg, Denmark

**Keywords:** Vocational school, Young students, School dropout, Intervention development, Ethnographic research, Field study, Social practice

## Abstract

**Background:**

This article describes the rationale and contents of an intervention program aimed at strengthening students’ social relations in order to reduce dropout from vocational schools in Denmark. Taking its theoretical cue from the concept of ‘social participation’, a qualitative study was performed to investigate the specific relationships between the social environment within the schools and the institutional structures in order to analyse reasons for school dropout and their relation to well-being, cigarette smoking and substance use.

**Methods:**

The development study was based on ethnographic methods, including 22 qualitative interviews with students 17–19 years old and fieldwork with participant observations at four vocational schools over 40 days, including informal interviews and discussion meetings with managers, teachers, counselors and students. As part of the fieldwork, four additional qualitative interviews and four group interviews were conducted with students 16–25 years old.

**Results:**

The qualitative data collection resulted in seven major themes to be addressed in the intervention: social relations, sole focus on professional skills, institutionalized individualization, importance of the introduction period, physical surroundings and schedules, tobacco and cannabis use and communication about drug use. The program addressing these themes incorporates suggestions that are meant to improve how teachers welcome new students, to enable greater integration of social and educational activities and to enhance the capacity of teachers and counselors to deal with drug use problems among students.

**Conclusion:**

The development of new intervention programs might benefit from adopting a theoretical and methodological perspective that enables a closer exploration of the everyday social practices in which interventions are embedded. Thus, we aimed to create a comprehensive intervention that worked through organizational changes in everyday school practices. Intervention programs must be planned in dialogue and collaboration with practitioners in the field to ensure the pertinence and usability of the program.

## Background

This article describes and discusses the rationale and contents of an intervention aimed at improving students’ social relations in order to reduce dropout from vocational schools as well as to achieve positive effects on students’ smoking and drug use. The study is based on ethnographic fieldwork within four vocational schools in Denmark and, taking inspiration from the theoretical concept of ‘social participation’ [1, 2], seeks to identify concrete opportunities for intervention that may strengthen students’ participation and relationships within the school setting.

In Denmark, only half the students enrolled in vocational training complete the program, and 40 % of those who drop out do not continue in any educational program within 10 years [3–5]. Compared with their peers in general upper-secondary school (both equivalent to the high school level), students attending vocational schools in Denmark have a greater risk of developing harmful patterns of tobacco, alcohol, and drug use [6, 7]. Gaining insight into the underlying causes is therefore important in order to prevent social marginalization and promote health and well-being among this particular group of students.

In relation to dropout, previous research has shown that relationships between students, relationships between students and teachers and the organizational setting of school life are important factors determining whether students complete educational programs. These studies indicate that developing positive social relations can be an incentive for students to attend school. This applies even to students who report that schoolwork is difficult and the demands are hard to meet [8–12]. The more students interact—not only with each other but also with teachers—the more likely they are to stay, especially in difficult periods of life when students have an imminent risk of dropping out [11]. Quite clearly, students stay in school when social relations with their teachers are positive. This association persists even when students’ background, school demographics and school sector are taken into account [6]. Thomas [13] emphasizes that the beneficial effects of staying in school must outweigh those of dropping out of school. Moreover, Tanggaard [14] and Thomas [13] emphasize students’ need for a sense of belonging as an important factor for remaining in school. Part of this sense of belonging seems to come from the certainty in one’s future based on having a supportive social network.

Although developing positive social relationships among students and teachers seems to be a protective factor against dropout, most research on school dropout has focused on students’ behaviour and performance in school. Little attention has been given to the influence of the schools themselves—their organization, management and teachers—on students’ decisions to drop out [15]. Beekhoven and Dekkers [16] argue that it is time to stop focusing on individual students and their backgrounds in relation to the causes of dropout and start looking at the schools themselves. Beekhoven and Dekkers [16] claim that the role of schools in pushing students out is underestimated. Both qualitative and quantitative studies report lack of social support by teachers as an important reason for dropping out: unengaged students say that teachers do not care about them, are not interested in how well they do and are not willing to help them with problems [8, 17–20]. Researchers thus indicate that how school life is organized, including the governance, curricula and educational methods, influences the completion of educational programs and students’ experiences of success in school participation [13, 16]. In addition, research [21–23] has shown that a school culture that is appropriate for students as young adults and an organization that is flexible and supportive help to re-engage students [24].

In relation to well-being and health behaviour, substantial evidence indicates that peers are an important social factor [25]. Some researchers pinpoint participation in peer groups as a major factor explaining substance use [26]. However, the relationship between social networks at school and young people’s substance use is complex. Substance use plays a role in becoming socially integrated [27, 28]. Nevertheless, evidence suggests that students often use substance use as a coping mechanism if they feel marginalized [29]. Lynskey and Hall also report that using such substances as alcohol, cannabis and other drugs influences whether students complete educational programs or drop out [30].

In Denmark, research [31–33] has shown that substance use plays a crucial role in forming peer relationships. Alcohol use is widespread and is, among other things, used to reinforce the establishment of social relationships. Cannabis remains illegal but is still widely accepted in certain groups of young people [34, 35]. Other illegal drugs are used much less, but substance use generally serves as an important marker and maker of social identity [7, 36]. This is especially important in transitions between educational institutions, such as from lower-secondary school to upper-secondary school [37, 38] or from upper-secondary school to university [39, 40]. In these periods, young people experience intense and demanding changes in their social life, and changes in their substance use often mediate and accompany these life changes. The role of tobacco and substance use and their relationship with educational transitions have not, however, been examined in the context of vocational schools, which differ from upper-secondary schools and universities in many ways. Associations have been found between students’ substance use and lower educational aspirations, lower educational expectations and increased likelihood of dropping out of educational programs [41, 42]. Thus, it can be concluded that social integration is strongly associated with substance use, that this has implications for adolescents’ educational progress and that the school environment plays a major role in this association [41]. This is worrying, because education is inherently an important determinant of health [43].

Despite ample research evidence of the significance of social and organizational conditions for educational attainment and the well-being of young people, little is known of specific intervention programs that may be useful in vocational schools in Denmark. This article aims to describe the rationale and contents of an intervention program that was developed in order to ameliorate this knowledge gap. We begin by providing some background information about the specific setting of vocational education in Denmark. Next, we outline the theoretical rationale and the methods used in the field study. Then we present key empirical findings from this exploratory study. Finally, we present the content of the intervention program that was developed in cooperation with the management, teachers and counselors at the four schools. We conclude by presenting some more-general reflections on developing an intervention within this type of setting.

### Vocational training in Denmark

In Denmark, about 19 % of each youth cohort obtains a vocational education, with 117 institutions offering basic vocationally oriented educational programs [3, 4]. Vocational education and training includes agricultural, commercial, technical, and social and health care programs. Denmark has 108 vocational education and training programs leading to jobs such as carpenter, electrician, mechanic, farming assistant, social or health care worker and hairdresser. They typically start with an upper-secondary basic course. About one third of the students in basic courses are 15–17 years old and half are 18–24 years old. To continue within the main programs, students are required to have a training contract for an apprenticeship. The main programs are alternating programs, in which practical training in a company alternates with teaching at a vocational school. The main programs vary in length; generally they take about 3–4 years [3, 4].

Since most dropout occurs during the basic course [4], we focused on the basic course in developing the intervention. There are no admission requirements, and each student receives an individual transition plan based on an individual competence assessment. The basic course is flexible in duration and depends on the individual student’s qualifications and ambitions. Students who have a contract for apprenticeship can take a targeted and shorter basic course. Students who need to refresh their previous qualifications and knowledge from primary and lower-secondary school or need additional time to complete the course can extend their basic course. A basic course typically lasts 10–40 weeks. Uptake to the basic course is normally ongoing. This means that students not only enter at different times but they work at their own pace. A student who enters after another student might in fact finish the basic course before the old student. Since new students enter the course continually, a class is not a definite unit but a group of students individually working on various tasks and assignments. In addition, because obtaining an apprenticeship contract is difficult, many students decide to transfer to another vocational program before they complete the course they started.

Vocational schools have several educational guidance counselors who support and guide the students in completing their education and training courses. The students are also assigned a contact teacher, who is supposed to contribute to a good educational environment and support each individual student. Some students with special needs might receive support from a mentor. In special cases, they can be offered psychological support [3, 4].

Research focusing on educational dropout in Denmark [14, 44–47] indicates that educational programs within Denmark’s vocational education system have become too individualized,^1^ and this affects dropout. Because of this individualization, in which each student follows an individual learning trajectory, the highly important sense of belonging to a community of students has been dissolved [48]. According to Koudahl [47], the social environment, which is especially important for students at risk of dropping out, has been impaired. Helms Jørgensen [46] states that the flexibility and the individualization within vocational education have contributed to a weakening of the social bonding. This affects students’ attendance and dropout. In a survey of the learning environment in Denmark conducted by the Danish Centre for Educational Environment, Helms Jørgensen [46] states that including students in learning communities with other students is the most important factor for retaining students within vocational education. In addition, a report by the Danish Evaluation Institute [49] states that creating communities for students across different professions contributes to developing a well-functioning learning environment. Thus, in this article a focus on students’ possibilities for participation within the vocational school context is our point of departure.

### Theoretical framework

The study takes its main theoretical cue from the concept of ‘participation’, which is rooted in social practice theory [50] and in critical psychology [1, 51]. In this line of theorizing, understanding people is a matter of focusing on how they live their everyday lives across different contexts of social practice [1, 52]. Social practice theory emphasizes the historical production of people in practice and pays attention to differences among participants and the ongoing struggles that develop across activities around those differences [53]. It emphasizes that, whether acting on their own or not, individuals participate in structures of social practice [1]. People are conceived as embedded in larger social, historical and cultural practices, participating in ways that are both structuring and structured in specific contexts of social practice. In critical psychology and in social practice theory, participation is a key concept. Applying this theoretical concept focuses on what people participate in, how they participate, with whom they participate, where they participate and for what personal reasons and following which interests and orientations they participate [1, 2].

Thus, the main theoretical assumption in the study was that students develop their participation and their habits in communities of social practice [50]. We understand habits broadly, including health-related habits such as smoking tobacco and cannabis and drinking alcohol, school attendance and study habits. Based on this theoretical framework, we constructed the vocational schools analytically as communities of social practice that provide varying possibilities for social participation. We assumed that participating in school practices transforms and develops students’ habits, which are deeply embedded in their relationships with peers and teachers. In this theoretical understanding, participation and developing habits are social matters [38, 54, 55]. Further, we assumed that student participation and formation of habits are also connected with the institutional arrangements of the schools. Consequently, considering interventions among students requires considering that various institutional arrangements frame their participation in the school setting and therefore condition the development of their social relations [38, 56–58].

## Methods

To gain insight into the vocational school context and to build knowledge about existing as well as potential possibilities for social participation in vocational schools, we adopted an exploratory method comprising qualitative interviews with students followed by ethnographic field studies at vocational schools, including informal interviews with management, teachers and counselors and additional interviews and group discussions with students. The research literature provides knowledge about dropout rates and the extent of substance use, but we had little knowledge about the specific organization of Denmark’s vocational schools and the implications for students’ social participation and educational attainment. We knew that many researchers had reported problems associated with individualization, but we wanted to more deeply understand how this challenge appeared in daily school practice, how it differed between school departments and between students’ future professions and how it might differ between students. Thus, the aim of this development work was to examine existing practices in order to identify concrete ways in which students’ social participation and relations may be strengthened.

The exploratory study was initiated in summer 2009. We conducted 22 qualitative interviews [59, 60] with students^2^ 17–19 years old (15 boys and 7 girls) who had entered vocational education two years earlier. Fourteen of these students had continued their vocational program, four students had transferred to general upper-secondary school and four students had dropped out and were either working or unemployed. The interviews were conducted as an interactive process [61, 62] with open-ended questions, guided by an interview guide. These interviews dealt with the students’ perceptions of the new school life and forms of participation and included their experiences with using tobacco and alcohol and other drugs. The interviews also dealt with the students’ participation in social life outside the school context: for instance, with whom the students associated in the evenings, where they met and what they did. Thus, the empirical material provided students’ specific descriptions of their participation in the new school setting and their everyday life. As these exploratory interviews provided a preliminary insight into associations between students’ social relations and their drug use only [35, 63, 64], we chose to explore the issue further through ethnographic fieldwork. This field study was carried out in order to provide more-specific insight into particular vocational school organizations and students’ participation, including development of social relations and students’ well-being as well as drug use leading to drop out.

From January to June 2010, three of the authors, two anthropologists and a psychologist, conducted ethnographic fieldwork at four vocational schools. The four schools are located in major towns dispersed in Denmark and offer a wide variety of education programs. The schools were chosen for participation through convenience sampling [62]. Colleagues in the municipalities where the schools are located established our contact at two schools. One of these schools established contact at a third school by recommendation, and a fourth school called and offered to participate in the study. However, in order to strengthen the generalizability of the findings, we pursued a maximum variation strategy [62] within the sampled schools by including participant observations in various educational programs (carpenters, bricklayers, painters, electricians, mechanics, cooks, graphic designers, and sign writers). The fieldwork was designed as an exploratory field study in a broad ethnographic sense and included 40 days of participant observations [65–67] with an equal distribution between the four schools and an equal distribution between new program starts and everyday practices. The included educational programs represent various professional traditions, teaching curricula, educational program length and ways of organizing the learning environment, as well as teachers and students with specific—but sometimes very different—interests related to their profession. The theoretical concept of participation guided the observations, as well as the following qualitative interviews. Thus, the focus in observations and interviews was *what* the students participated in, *how* they participated, *with whom* they participated, *where* they participated and for what *personal reasons* and following which *interests* and *personal orientations* they participated [1, 2]. More specifically, the participant observations focused on the professional practices at the schools as well as the students’ social interaction and their participation in social activities in the school. The participant observations also included a focus on the students’ *conditions* for school participation and the *organization* of specific professional practices, physical arrangements, routines, school traditions, ideologies, social norms, professional self-perception, the schools’ demands of the students and students’ expectations for other students.

We took field notes while present in the field. The notes captured what was said, what happened, the atmosphere, descriptions of contexts and places, specific activities, the persons present and what they were doing. The notes were afterwards written into expanded notes (300 pages). These notes varied from small notes to thick descriptions [68]. Informal conversations with students (ranging from 16 years old to middle-aged) took place daily as part of the participant observations. These conversations dealt with the students’ perceptions of their participation within the school setting as well as outside the school and included their use of tobacco and alcohol and other drugs. These informal conversations were followed by four additional in-depth qualitative interviews with individual students and four group interviews with two to three students 16–25 years old in each group. These interviews and group discussions were conducted to explore certain incidents, reactions and questions that had occurred during the participant observation period. All interviews were transcribed word for word.

According to Bartholomew [69], researchers must involve practitioners, who shall later implement the intervention, in the preparation phase.^3^ The participant observations were therefore followed by informal conversations and several discussion meetings with managers, teachers and educational guidance counselors. These discussions focused on their assumptions about students’ attendance and dropout, social and mental health problems and problems with the use of tobacco and alcohol and other drugs. In addition, the discussions concerned the school staff’s conceptions of the needs and expectations of the young people attending their schools and considerations about whether the organization of school practices and curricula generally met and supported the students’ expectations and specific needs.

Profoundly knowing the specific context of vocational schools is a prerequisite for developing an intervention within this setting. The collaboration with the school management and staff continued into the more specific planning of the intervention program. This continual collaboration with the actors in the field was a precondition for developing an intervention program sensitive to the distinctive and complex school practices of vocational schools, as other studies [70–72] have shown. Including the students in this part of developing the intervention program would have been highly relevant. However, according to the school management, the short duration of the basic courses made the students’ participation impossible as this would have conflicted with the students’ focus on the curriculum and their acquisition of professional skills.

We presented preliminary outlines of our analysis to the school staff at several meetings at each of the four schools. The intervention program was planned to be a tool for management and teachers, and involving them and giving them the opportunity to put their fingerprint on the suggested intervention was of utmost importance. Based on the feedback we received at these discussion meetings, we reformulated the program to match the specific conditions within the field. Thus, dialogue between practitioners and researchers determined not only the findings of the exploratory fieldwork, including our knowledge about the students’ perspectives on their new school life, but also the development of the intervention program.

The material from interviews and fieldwork was first coded in NVivo, adopting an inductive approach and basic principles of cross-sectional indexing as described by Mason [73]. Through an iterative process, in which theories of social practice and participation served to guide our readings of the data, we developed the codes and categories into seven overarching themes, which we use to structure our presentation of results.

### Ethics

The Danish Data Protection Agency has approved the study. We informed management, teachers, counselors and students about the purposes of the study and the methods. Consent was obtained formally through agreements with the school management, which provided us with access to the school facilities and particular members of the staff who served as key informants. We obtained consent from other staff members and students through an ongoing dialogue in which we stressed that their participation in the research was voluntary. We also emphasized that they were free to withdraw from the research at any time. In qualitative and ethnographic research, ethics is an embedded, continuous concern acknowledging how the researcher’s presence in the field of research is a unique, essential point of knowledge making [74].

Ensuring that all information would be treated confidentially was of utmost importance for developing trust between the researchers and students and between the researchers and the teachers and managers. In the daily communication between the researchers, students, teachers, counselors and managers, no comments or information gained from one informant was passed on to other informants in the field. All participants are anonymized in this article.

## Results and discussion

### Key findings

We present key findings from the exploratory research that provided the basis for developing the intervention and its specific program components.

#### Social relations and new friendships are important

Students starting a vocational education program expect to develop social relationships. The youngest students (15–16 years old), who came directly from lower-secondary school, especially emphasized this perspective in the interviews. However, vocational schools have no traditions for school gatherings, parties, café evenings, debate sessions or music events. The students have to organize social life when the school day is over. This is in contrast to upper-secondary schools (with students in the same age group), in which such organized social gatherings are very common. This nurtures certain expectations among students attending vocational schools, and they often complain about the lack of social activities. Two students said:

I thought there would be some get-togethers at the school. A Friday bar or café or something. But the school had no social events we could join.

This is fucking ridiculous! To put it mildly! We were all royally pissed about this! There are never any parties here—and we cannot get into the parties at the upper-secondary schools as craftspeople.

According to the students, school-related friends are people they can see every morning and spend time with during the school day; work with on school projects; discuss professional matters with and learn from; and take a break with, have fun with, meet when school is over and party with during weekends. However, peer groups and friendships change when students enter a new educational program, and especially the young students starting vocational school want specifically to develop new social relations, including making new friends within the new school setting.

The group of young students who came directly from lower-secondary school seemed to consider the general lack of priority on developing social relations in the school context to be a problem. The interviewed students who had dropped out of vocational school reported disappointment with the social environment within the schools as a main reason that had led to dropping out. In contrast, most students older than 25 years had families and an established circle of friends and therefore had a less pronounced need for new friends. However, all students said that they valued positive relations within the school context.

#### Schools focus on professional skills, not social relations

Vocational schools do not tend to give priority to developing social relations among the students, and teachers appear to disagree on the importance of social relations for learning. Some teachers say that the main task of vocational schools is developing students’ professional skills to enable them to meet the demands of their future apprenticeship. These teachers do not link developing students’ professional skills to developing social relations. A teacher explains:

We—here at this type of school—focus on the schoolwork and not so much on the social relationships. Craftspeople are efficient and resist education. We do not have time for grand educational theorizing.

Other teachers explain that the short length of the basic courses (in general, 10–40 weeks) contributes to the lack of focus on developing a more committed social environment among the students:

It is hardly worth focusing on the students’ social relations because the basic course period is very short.

However, during the discussion meetings with the teachers, we also realized that the teachers have intensely debated this attitude recently. Some teachers argue that focusing on developing positive social processes is required for developing the students’ learning processes. At one of the schools, this led to an intense debate among the teachers after one teacher took the newly started students outdoors to sit on the lawn—having a group talk about what was going on in their everyday life—as a way of socializing and getting to know each other. A teacher said:

Some of my colleagues protested vigorously, because what is the purpose? Why are you doing that when the students are attending school? We have to focus on the coursework. The other things are bullshit. They have nothing to do with instruction.

Discussion meetings with the teachers revealed that, regardless of this ongoing debate among the teachers, the general practice at the four schools is that school life is not organized to ensure the development of students’ social relations, either during the introduction period in the first few days or in everyday practice.

#### Institutionalized individualization

The organization of a school day within vocational schools has distinctive features significantly different from what the students experienced in primary and lower-secondary school. Although students within vocational schools sometimes work in groups, they primarily work individually according to an individual education plan. According to the teachers, students’ maturity is a precondition for autonomously planning and administering the school day and developing learning processes. The older and more mature students especially share this viewpoint, since they are more capable of planning the school day on their own. However, many students do not have such competencies when they enter vocational school. When asked in the interviews about the main differences between lower-secondary school and vocational school, many young students point out that in vocational education, “you take a break when you feel like it”. Especially in the arts and crafts professions, students work on individual tasks, at their own pace, depending on their individual competencies and personal school motivation and orientation. Although the school day, according to the schedule, is organized into instruction time and break time, some students—because the learning processes are individualized—experience considerable freedom to decide when to “take the liberty to have a break”. According to the teachers, this liberty is very much appreciated, and these breaks very often become arenas for developing social relations. Sometimes the students meet for a cup of coffee in the cafeteria. However, cigarette smoking outside the workshops is a common arena for developing social relations.

#### Rules or socializing? The introduction period is important

Since basic courses start two, four (most typical) or six times a year, receiving new students seems to be common. According to our participant observations, the introduction period very often focuses on registering the new students, enabling them to access the school computer network, assessing their professional and academic competencies to divide them into classes according to their academic qualifications, a hasty orientation about the timetable and the curriculum, general information about protective clothing and rules about smoking, using alcohol and other drugs and attendance.

Many schools had no events at all focusing on helping the new students get to know each other. A student interviewed during the lunch period on his fifth day within the new educational setting was sorely disappointed:

Just imagine, we have not even learned each other’s names yet.

Students in this case experienced an introduction focusing on formal school rules, especially a pep talk about the rules for school attendance, including the strict rules and consequences if they do not arrive in class on time.

Our participant observations indicate that a few departments may be exceptions to the rule. Here the new students are welcomed with a festive reception characterized by welcoming speeches, a round of introductions in which both teachers and students present themselves and perhaps a presentation of the older students’ projects so that the new students get an idea of the profession they are about to enter and the professional competencies they will acquire during the coming weeks. According to the teachers, this kind of introduction means that the students can experience the first day as a festive occasion, they can get to know each other, they can learn what they will be taught during the basic course and they can start to develop a professional identity.

#### Confusion and insecurity related to physical surroundings and timetables

The general lack of focus on developing social relations within vocational schools is reflected in the physical surroundings. Some schools have no special places for social gatherings: that is, no halls, small sites or cosy niches organized for students across different classes to meet during breaks and develop their relations and friendships. Some schools are former industrial buildings and were not originally designed for educational purposes. Our participant observations and discussions with the counselors revealed that new students sometimes have difficulty finding their way to the right destination within the new school location. In general, vocational schools are huge institutions with many sections—and newcomers do not always find it simple and easy to navigate their way to the classroom or workshop.

In addition, the timetable the students receive is very often a rough printout from the information technology system. Instead of clear and detailed information on the course, the location, the exact meeting time and the specific clothing required for participation in the course, the timetable information is almost always presented in abbreviations that are not easy to understand. The students are therefore very often confused and insecure; according to the counselors, this might contribute to truancy and dropout. However, some school departments give the students a clear timetable with a clear-cut description of the course, the meeting time and the clothing requirements, and the teachers review the curriculum to introduce the course content and the schedule of the basic training. According to both teachers and counselors, this leads to students knowing when and where to attend in the following days and what clothing to wear. During the fieldwork, we also observed that some departments organized preliminary meetings with students and their parents before school started. These meetings included a guided tour of the school and a presentation of the school facilities, including a description of the profession, to give both the future students and their parents a first impression of the educational program. According to the teachers, these meetings give parents the opportunity to meet a teacher or a counselor and to learn whom to contact in case of problems.

#### Tobacco and cannabis use as reported reasons for dropping out

The students’ participation in cigarette smoking communities is closely linked to making new social relationships. According to some students, the smoking areas are the cosiest places on the school grounds. These areas are where relationships develop, not only within the students’ own classes but also across educational programs. Around the ashtray, students from the painting department or the hairdresser program can meet with students from the auto mechanic or the carpenter programs. In the interviews, some students reported that taking up smoking when starting at the vocational school was an easy way to socialize. However, not all students want to start smoking. Interviews with nonsmoking students show that being a nonsmoker can lead to being excluded from the relationships developed between the smokers. Since these smoking relationships sometimes include both teachers and students, the nonsmokers sometimes have to wait to receive a teacher’s guidance and attention.

Participation in cigarette smoking communities to develop social relations and friendships is not unique to students within vocational schools. Students within other types of youth education in Denmark mention joining the smoking group to develop new social relations. For some students, however, the number of breaks increases over time. From the perspectives of the teachers—and some students as well—breaks taking place besides the scheduled breaks interrupt both the rhythm of the school day and the students’ concentration on acquiring craft skills. The focus drifts away from learning professional competencies towards social interactions centring on tobacco and, for a small group of students, cannabis. This has severe consequences for this group of students. Teachers, counselors and students describe how the management challenge of balancing time and focus between assignment work and self-administered breaks might lead to problems in passing the examination, and thus to an extension of the education period.

#### Identifying and addressing problems related to substance use

Conversations and interviews with teachers and counselors showed that they had difficulty in identifying and addressing problems related to students’ use of alcohol and other drugs. Teachers and counselors regularly experience students whose attention drifts away from coursework and school life in general, and in some cases they suspected that one reason was the use of drugs (especially cannabis). They felt, however, that their knowledge of potential solutions to this problem was limited.

Most schools have adopted strict zero-tolerance policies prohibiting the use of both legal and illegal intoxicants during school hours. In one school, a specially trained dog was used to search for cannabis in students’ belongings. This was meant to have a preventive effect, and to enforce the zero-tolerance policy. However, teachers and counselors did not always consider it appropriate to expel students for possessing or using drugs. During a visit, we witnessed a student being escorted out of the school by a security guard who had found cannabis in the student’s bag. The student’s counselor told us that she had worked hard the preceding months to support the student and that he was very close to completing his basic course. In that particular case, the zero-tolerance drug policy conflicted with the overarching aim of supporting students in completing their education. In general, we identified a need to develop new solutions to problems related to substance use among the students.

### The intervention program

The exploration phase aimed to analyse the existing institutional arrangements and their implications for the development of students’ social relations and well-being, as well as smoking and the use of alcohol and other drugs leading to dropout among the students. Based on this exploratory work and the underlying theoretical perspective, we identified specific opportunities for the intervention. The process of translating the key analytical findings into specific suggestions for the intervention program took place in close collaboration with school managers and teachers who helped us structure the suggestions into three main areas and to phrase them in terms that were perceived as easy to understand and implement.

As described in the key findings, our analysis identified several examples of practices that seemed to work well for staff and students in particular schools. Our observations during fieldwork indicated that these ‘good examples’ could lead towards the overall aims that we wished to achieve with the intervention. Further, these practices were in accordance with our theoretical perspective focusing on participation and the development of social relations in a school context. These good examples were the main subjects for our discussion meetings with the practitioners. The program is thus to be seen as a collation of the good examples we identified through fieldwork in the schools, and which we hypothesize may contribute to strengthened social relations and reduced school dropout.

**Fig. 1:**
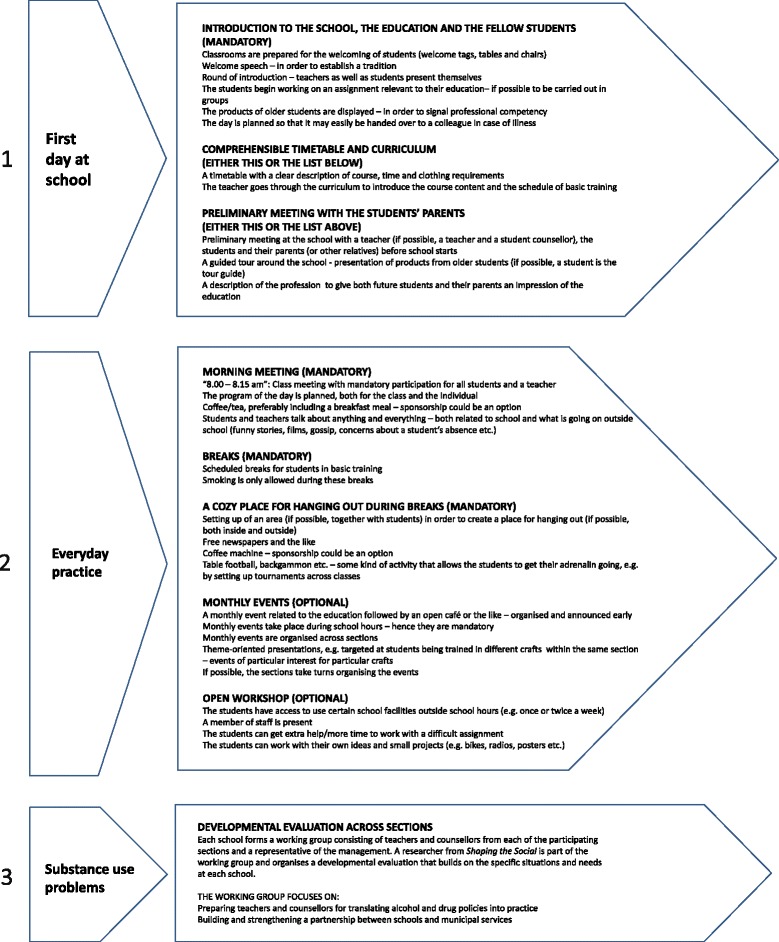
Intervention program

The intervention program presented in Fig. 1 is a tool to be used by school management and teachers to initiate activities in three areas:

1. First day at school: here the program focuses on introducing students to the school, the educational program, the profession and the fellow students.
2. Everyday practice: here the program focuses on changing everyday practice to integrate social activities with professional learning.
3. Problematic situations related to substance use: here the program focuses on activities that enhance the capacity of the staff to deal with problems related to substance use.

Below, we present the suggestions in each of the three areas in more detail. It should be noted, however, that the program, as presented in Fig. 1, does not include a manual or more detailed instructions related to the implementation of the suggestions, because we wished the program to be adapted and elaborated by the school staff.

#### Activities on the first day of school: introducing students to the school, the educational program, the profession and fellow students

The aim of this part of the program is for students to get to know each other and to feel welcome. The specific suggestions are based on our finding that introduction periods are important opportunities for the formation of social relations. We also found that, even though schools tend to focus on practical issues when welcoming new students, they often find it difficult to manage the logistics involved in their new school life. For these reasons the program included the following specific recommendations: The introduction to the school should be experienced as a festive occasion. Receiving a clear timetable and knowing the location of classrooms helps students get to class on time and in appropriate clothing. Knowing early in the course what they will be taught during the basic course might help to enhance the development of their professional identity. Parents who know the school demands and the school personnel can better support the students.

#### Activities in everyday practice: morning meetings, breaks, a nook for hanging out, monthly events and open workshops

We found during fieldwork that the schools focus on professional skills rather than social relations, even though students find it important to develop new friendships. The suggestions in this part of the program are based on the basic idea that the development of social relations needs to be closely tied to the educational aims of the schools. Thus, the program suggests five forms of changes in everyday practice to integrate the establishment of positive social relationships with learning and coursework. The morning meetings aim to strengthen the relations between students and teachers; students gather around a joint social activity, and for each student the curriculum for the day is planned. The scheduled break allows students to concentrate during lessons. When all students take breaks at the same time, they have more fellow students with whom they can get acquainted. The cosy place for hanging out during breaks aims to strengthen students’ social ties: they know where to go to hang out with others and have an alternative to the smoking areas. In addition, organizing arrangements for students across professions contributes to developing a well-functioning learning environment. The monthly events and the open workshop enable students to develop a professional identity and social ties. Students should get to know each other across sections because the more people they know, the more fun it is for them to show up every day and be educated. In addition, students can pursue their own interests and socialize around a professional activity of their own choice, inspire each other and develop social ties related to their future profession.

#### Activities regarding substance use problems: problematic situations related to substance use

This intervention element aims to enhance the capacity of the staff to deal with problems related to substance use. We found during fieldwork that many staff members did not feel qualified to identify and address substance use among their students, and that they felt a need to develop new skills and/or procedures in this area. The program suggests that each school forms a working group comprising teachers and counselors from each of the participating sections and a representative of the management. A researcher from the project group is part of the working group and organizes a developmental evaluation [75] based on the specific situations and needs at each school. The working group focuses on preparing teachers and counselors for translating policies on the use of alcohol and other drugs into practice and for building and strengthening a partnership between schools and the municipal services.

## Conclusion

In line with earlier work in this field [8–10], our study suggests that the reasons for dropping out of vocational education are numerous and complex. Some students drop out because of social and personal difficulties that result in a general lack of well-being. Some students drop out because their school performance is poor or because they perceive their future prospects as poor whether or not they finish their educational program. Some students give priority to time spent with friends centring on cigarette smoking and, for some students, cannabis. Some students drop out because they are bored, although they are doing well in school. Some students drop out because they are not able to get an apprenticeship agreement when they pass their basic course examination. There is no “typical” dropout, as Rumberger [15] states. In his review of issues and evidence related to dropout, he emphasizes that understanding the causes of dropout is complex. Dropping out of school might be viewed as a process of disengagement from school, perhaps for either social or academic reasons, which culminates in the final act of leaving. Developing a basic understanding of these school processes before developing an intervention program is therefore important [15, 76].

In this study we adopted a theoretical approach which stresses the importance of participation in communities of social practice [50]. This approach guided our observations in the field and served as an important source of inspiration when designing the intervention. Our experience was that our theoretical concepts and ideas resonated well with the experiences of school staff members as well as students. To our knowledge, no prior studies have adopted this particular theoretical approach in public health intervention research, but our study suggests that it can be a useful framework for intervention development.

Our study shows that reducing student dropout calls for a multifaceted intervention program, including both new initiatives and changes at the organizational level and regarding teachers’ practices. The intervention thus aims to shape the conditions for the students’ development of social relations by reshaping the school structure regarding their participation in vocational school life. The basic idea is to integrate the development of positive social relationships with learning and training. As shown, this requires changing many practices in everyday vocational school life. Students’ experiences of positive social relationships promote general well-being within the school context and might lead to positive focus on school. In the longer term, improving social relations might influence the students’ completion of the educational programs and reduce the use of tobacco and harmful substances, improving general well-being and health.

However, this intervention program also presents challenges. The program is meant to improve how the teachers welcome new students, to enable greater integration of social and educational activities and to enhance the capacity of teachers and counselors to deal with problems related to the use of alcohol and other drugs among the students. These suggestions may be incorporated into the everyday practices at the various departments in many ways and call for an intervention program that enables flexibility for practitioners. In addition, an intervention can only be socially effective if it succeeds in creating a shared understanding between researchers and practitioners that enables the intervention to become implemented under ever-shifting social circumstances. Rod et al. [77] propose that the term “the spirit of the intervention” be used as an entry point for discussing the dimensions of an intervention that make it socially effective. We also propose that thinking of the intervention program as a set of principles that may be applied in the daily practices of the teachers can guide this discussion with the practitioners [77].

Future research will address the actual implementation of the intervention program and examine its effectiveness. Our findings indicate that a strong potential exists for strengthening students’ social participation and relationships in the context of vocational schools.

## Footnotes

1 Reform 2000 introduced increased individualization, flexibility and modularization of basic courses. The aim was to enable strong students to fast-track the basic course and to improve access from vocational education to higher education. In addition, an important aim was that weaker students could spend more time in the basic course to acquire the necessary competencies to continue to the main course. However, researchers’ analysis [46] has shown that the reform did not achieve its objectives – especially not for the weaker students.

2 These interviews were conducted with participants in the Ungeshverdag.dk cohort, who had been followed since grade 7 and through grades 8 and 9. In 2009, the participants started in youth education (either vocational school or upper-secondary school).

3 The four schools participated in the implementation of the final intervention program. The results of this intervention study are under preparation and will be reported elsewhere.
